# What Are the Neurotoxins in Hemotoxic Snake Venoms?

**DOI:** 10.3390/ijms24032919

**Published:** 2023-02-02

**Authors:** Alexey Osipov, Yuri Utkin

**Affiliations:** Shemyakin-Ovchinnikov Institute of Bioorganic Chemistry, Russian Academy of Sciences, 117997 Moscow, Russia

**Keywords:** azemiopsin, cysteine-rich secretory protein, hemotoxic venom, nervous system, nicotinic acetylcholine receptor, phospholipase A_2_, postsynaptic neurotoxin, presynaptic neurotoxin, three-finger toxin, viperid

## Abstract

Snake venoms as tools for hunting are primarily aimed at the most vital systems of the prey, especially the nervous and circulatory systems. In general, snakes of the Elapidae family produce neurotoxic venoms comprising of toxins targeting the nervous system, while snakes of the Viperidae family and most rear-fanged snakes produce hemotoxic venoms directed mainly on blood coagulation. However, it is not all so clear. Some bites by viperids results in neurotoxic signs and it is now known that hemotoxic venoms do contain neurotoxic components. For example, viperid phospholipases A_2_ may manifest pre- or/and postsynaptic activity and be involved in pain and analgesia. There are other neurotoxins belonging to diverse families ranging from large multi-subunit proteins (e.g., C-type lectin-like proteins) to short peptide neurotoxins (e.g., waglerins and azemiopsin), which are found in hemotoxic venoms. Other neurotoxins from hemotoxic venoms include baptides, crotamine, cysteine-rich secretory proteins, Kunitz-type protease inhibitors, sarafotoxins and three-finger toxins. Some of these toxins exhibit postsynaptic activity, while others affect the functioning of voltage-dependent ion channels. This review represents the first attempt to systematize data on the neurotoxins from “non-neurotoxic” snake venom. The structural and functional characteristic of these neurotoxins affecting diverse targets in the nervous system are considered.

## 1. Introduction

Snakes (Serpentes) in Linnean taxonomy form a suborder in the order Squamata from the class Reptilia [1]. There are over 3500 species of snakes [2]. Snakes are legless predators and use a variety of hunting strategies. In practice, some snakes subdue and swallow large, often dangerous prey animals by constriction or using strike-and-release feeding strategies; this shields them from injuries during predatory encounters. Others use venoms to immobilize preys. Snake venom has evolved from saliva in the course of evolution. It is produced in a special organ venom gland and is delivered at the bite through the teeth that are often referred to as ‘fangs’, which have a channel or groove for the injection of venom. Various sources indicate different numbers of snake species which are considered as venomous. At the time of writing the review (the beginning of 2023), the most complete list of venomous snakes, including about 600 species, was presented on Wikipedia [3]. The World Health Organization (WHO) considers that about 300 species, mainly belonging to the families Viperidae, Elapidae and Colubridae, to be of medical importance [4]. It is believed that the evolution of snake venom is driven by an evolutionary arms race between venom toxins and prey physiology [5]. Since venom is an important functional feature of venomous snakes, its composition and activity has evolved in parallel with the physiology of prey [6]. Apparently, depending on which of the systems (nervous or cardiovascular) of the victim is most vulnerable, the venom was specialized as either neurotoxic or hemotoxic. In addition, the composition of venom depends not only on snake species, but also on some other factors, including habitat, season, age and size of the snake etc.

Snake venom is a complex lethal cocktail, composed mainly of peptides and proteins usually named “toxins” and targeting different systems of prey organism, including the nervous and cardiovascular systems [7,8]. Venoms that have a principal damaging effect on the nervous system or blood are considered as neurotoxic or hemotoxic, respectively. Most venomous snakes belong to the elapid or viperid families. In general, the elapid venoms comprise toxins affecting the nervous system and are considered as neurotoxic, while the action of viperid venoms is directed mainly on blood coagulation and they are regarded as hemotoxic. The venoms of kraits, mambas and most cobras are typical examples of neurotoxic venoms. The typical hemotoxic venoms are those of saw-scaled (carpet) vipers, Levantine viper, and most pit vipers, although the venoms of some pit vipers manifest neurotoxicity. However, the division into neurotoxic and hemotoxic venoms is entirely arbitrary because those from elapid may impair blood coagulation and, vice versa, the venoms from viperid can produce neurological signs. Interestingly, snakes from the Colubridae family, or rear-fanged snakes considered for a long time as non-venomous, are now recognized as venom-producing and include both species, which produce venom with prevalence neurotoxic components, such as elapid, and the species with hemotoxic venoms, such as viperid [9].

Components directly affecting the nervous system, in particular α-neurotoxins, β-neurotoxins, dendrotoxins, and some others, constitute a considerable part of neurotoxic (e.g., elapid) snake venom. According to proteomic data, α-neurotoxins may comprise more than a half of total proteins in some cobra venoms. For example, the venom of monocled cobra (*Naja kaouthia*) contains more than 53% α-neurotoxins [10] and Samar cobra (*N. samarensis*)—more than 65% [11]. Proteomic analysis revealed that β-neurotoxins called therein as β-bungarotoxins are among the main components of krait venoms. Thus, common krait (*Bungarus caeruleus*) venom contains about 13% of β-bungarotoxins [12], while in the venom of the many-banded krait (*B. multicinctus*) from Vietnam, the content of these toxins reaches up to 45% [13].

These toxins are responsible for both the predominant envenomation symptoms and the death of the victim. They have specific molecular targets, which are involved in nerve impulse transduction. For example, dendrotoxins target the presynaptic voltage-dependent potassium ion channels [14]. The α-neurotoxins disrupt the nerve impulse transduction at the nerve-muscle junctions and between neurons, primarily by blocking the reception of the mediator acetylcholine at postsynaptic nicotinic acetylcholine receptors [15].The β-neurotoxins act at the presynaptic site hampering primarily such an important process as the release of a mediator acetylcholine. Hence, in the narrow sense, neurotoxicity can be defined as a specific action on a molecular target within excitable tissue (it may be a membrane receptor or an ion channel) that affects directly the generation, transduction and reception of a nerve impulse. Based on this definition, a neurotoxin should not be obligatory highly toxic and lethal. For instance, neurotoxins, which disrupt nociception, sometimes manifest relatively lower lethality.

The components of hemotoxic venoms affecting blood coagulation are represented both by enzymes including serine and metalloproteinases, and non-enzymatic proteins such as disintegrins, as well as C-type lectin like proteins. According to proteomic data, these proteins dominate in viperid venoms. So, the venom of Nigerian carpet viper *Echis ocellatus* contains 30.84% metalloproteinase and 15.5% serine proteinase [16]. In the Nigerian puff adder *Bitis arietans* venom, these enzymes comprise 21.06% and 22.31%, respectively [16]. In the Saharan horned viper *Cerastes cerastes* venom, the content of disintegrins achieves 43.44% [17]. Hemotoxic viperid venom may affect the functions of the central nervous system by disturbing the blood clotting system and platelet aggregation, as well as by damaging vascular endothelium to cause severe intracranial bleeding and/or brain infarction [18]. Of course, this is accompanied by strong neurological disorders. Such complications are attributable to venom serine proteinases [19], metaloproteinases [20] and disintegrins [21], C-type lectin like proteins [22]. Should these compounds be considered as neurotoxins? Apparently, they should not, as they have no specific targets in excitable tissues. However, hemotoxic snake venoms do contain neurotoxins, i.e., compounds directed towards specific molecular targets involved in the propagation of the nerve impulse.

The neurotoxicity of snake venom is manifested mainly in the disruption of the neuromuscular transmission, primarily in the skeletal muscles. The explanation is simple: venomous snakes are predators who have neither claws nor powerful paws; but with victims’ skeletal muscle paralysis these are not necessary because a prey is unable to breathe, escape and resist. Therefore, the breakdown of movements is the leading strategy for the neurotoxins of snake venoms—both neurotoxic and hemotoxic [22]. The elements of the mechanism of transmission of an electrochemical impulse from nerve to muscle, which may be the targets for most neurotoxins of snake venom, are as follows: sodium and potassium channels of the nerve fiber; the release of the mediator from the pre-synaptic membrane; the passage of the released mediator in the synaptic cleft; the reception of the mediator by receptors on the post-synaptic membrane of the muscle; and the sodium, potassium and calcium channels of muscle fiber. With the specificity inherent in snake venom toxins, each neurotoxin usually affects only one such target. The neurotoxins present in elapid venoms affect practically all these targets, while viperid neurotoxins, being not so numerous, impact only some of the above targets.

Although the neurotoxic effects of the viperid venoms are well known and some neurotoxins have been isolated, there is still no review on viperid neurotoxins. This paper represents the first attempt to systematize data on the viperid neurotoxins.

## 2. Neurological Signs Produced by Viperid Bites

The venom of most viperids has a hemolytic effect; victims die from blood incoagulability and numerous hemorrhages in internal organs. However, neurological signs are often observed. The first documented report of neurotoxic manifestations after a viper bite that we could find dates back to the thirties of the last century. These were envenomings by the Serbian (Valley of the Sava River) *Vipera berus bosniensis* viper, described by Reuss as cited in [23]. 

The neurotoxic effects observed after the Russell’s viper (*Daboia russelii*) bites are well known. These effects were described mainly for Sri Lankan Russell’s viper and the neuromuscular dysfunction reported in patients was mild [24]. It was characterized by ptosis, blurred vision and ophthalmoplegia [25]; however, life-threatening paralysis was rare. 

Among European vipers, the bites by *V. ammodytes ammodytes* (the longnosed viper) can result in life-threatening neurotoxicity [26], and the venom of this snake contains several neurotoxins which are discussed below. As mentioned earlier, the neurotoxic manifestations are observed after the bites by Serbian *V. berus bosniensis*. Recently a severe neurotoxic envenoming following this snake bite has been reported in South-Western Hungary [27]. Neurological complications after the bites of snakes from *Vipera* genus have been reported in Italy [28] and Switzerland [29]. In south-east France, human envenomation after *V. aspis aspis* bites produces neurological signs (mainly cranial nerve disturbances) [30]. Using zebrafish as a model animal, neurotoxic effects has been demonstrated for *Montivipera bornmuelleri* venom [31]. 

All these data suggest the presence of neurotoxic components in viperid venoms and may indicate a multitarget strategy during envenomation. 

The venoms of snakes from the *Crotalus* genus produce neurotoxic signs in both humans [32] and animals [33]. Thus, after Mojave rattlesnake (*Crotalus scutulatus*) bites, respiratory paralysis may follow that suggest neurotoxic block [34]. In 1938, Slotta and Fraenkel-Conrat communicated the isolation of crotoxin from the venom of rattlesnake *C. terrificus*; this toxin became the first neurotoxin isolated from the viperid venom [35]. Crotoxin belongs to a family of secretory phospholipases A2, which we would like to start this review with.

## 3. Secretory Phospholipases A_2_

Secretory phospholipases A_2_ (sPLA2), as a rule, are small proteins of 12–19 kDa. The main characteristic of sPLA2 is hydrolysis of phospholipids at sn2-position in a Ca^2+^-dependent manner with releasing unsaturated fatty acid and lysophospholipid. However, sPLA2s are able to bind a lot of various proteins with relatively high affinity [36]. The active center of an enzyme contains Asp49, which may be substituted in enzymatically inactive homologues. Structurally and functionally, sPLA2s are now divided into 11 groups [37]. sPLA2s from Elapidae venom belong to group IA and sPLA2s from Viperidae venom belong to group IIA or IIB. sPLA2s from groups IA and IIA contain seven disulfide bonds six of which are conserved in both groups, while Cys11-Cys77 is present in group IA only and Cys51-Cys133 in group IIA. In addition, there is C-terminal extension of seven amino acid residues in group IIA. sPLA2s from the group IIB contains only six disulfide bonds, Cys61-Cys91 bond being absent, and C-terminal extension of six amino acid residues [38]. Several group II sPLAs form non-covalent dimers (rarely trimers or even pentamers) of homologous subunits. One of the subunits (usually basic) has enzymatic (phospholipase) activity while another (usually acidic) is enzymatically inactive and serves as its inhibitor and pharmacological modulator. The venom of one snake species may contain several homologous isoforms of sPLA2. So, crotoxin, earlier mentioned first neurotoxin isolated from viperid venom, is a heterodimeric sPLA2 from *C. durissus terrificus*. It comprises a basic crotoxin B (CB) subunit of 122 residues (represented by isoforms CBa, CBb, CBc, and CBd) non-covalently bound to an acidic crotoxin A (CA) subunit (represented by isoforms CA_1_-CA_4_) composed of three covalently linked polypeptide chains (α of 39 amino acid residues, β of 35 residues and γ of 14 residues), which are produced by proteolysis from a PLA2-like precursor. Crotoxin may consist of CA and CB subunits in all possible combinations [39,40].

### 3.1. Presynaptic Neurotoxicity

In snake venom, presynaptic neurotoxins, also called β-neurotoxins, are represented by sPLA2s of the groups IA and II, as well as by β-bungarotoxins, in which a sPLA2 subunit is bound by disulfide with Kunitz-type proteinase inhibitor subunit. β-Bungarotoxins are not considered here as they are found in the venoms of kraits from Elapidae family. β-Neurotoxins in hemotoxic viperid venom are present almost exclusively as the sPLA2s of group II.

Principal events at a presynaptic neurotoxin action on a nerve terminal have been comprehensively reviewed in [41], with updates [42]. These toxins disturb the release of a mediator from presynaptic site, and fundamental in presynaptic neurotoxicity is the phospholipase activity of the toxins, which leads to significant changes in the physicochemical properties of the membrane. Most snake venom sPLA2s are highly active enzymes, but not so many possess presynaptic neurotoxicity. So, presynaptic neurotoxins with different lethality do not differ substantially in their phospholipolytic activity. Moreover, levels of enzymatic activity and presynaptic neurotoxicity of sPLA2s do not correlate. Therefore, there should be some binding sites for presynaptic neurotoxins targeting these toxins to the presynaptic membrane, but a putative receptor is still disputable. For example, it may be N-type sPLA2 receptor, because the binding affinities of different sPLA2s to this receptor correlate strongly with their lethality. Nevertheless, products of phospholipolysis produced by presynaptic neurotoxins promote the exocytosis of synaptic vesicles, which enhances toxic effect. The conductivity of particular Ca^2+^-channels is also increased (perhaps, by presynaptic neurotoxins [43]) promoting the release of SNARE-mediated neurotransmitter and supporting phospholipase activity. The rapid internalization of the neurotoxins into nerve cells, including the terminal of a mammalian motoneuron [44], ensures fully expressed presynaptic neurotoxicity. Presynaptic neurotoxins probably exploit the synaptic vesicle recycling machinery to translocate across membrane; in the cell, they enter into the cytosol simply by hydrolyzing the vesicles. Then, these toxins bind some proteins in the cytosol and mitochondria, among which proteins 14-3-3 localize them inside the nerve ending. Calmodulin stabilizes presynaptic neurotoxins and increases their enzymatic activity in cytosol [41,42]; at the same time protein disulfide isomerase provides retrograde trafficking [45]. The binding to subunit II of mitochondrial cytochrome c oxidase may be responsible for the specific association of presynaptic neurotoxins with mitochondria [46], that leads to the opening of permeability transition pores and to their complete degeneration [42]. The enzymatic activity is unnecessary at least for the processes of the internalization of a presynaptic neurotoxin ammodytoxin, and its translocation into mitochondria [47]. Interestingly, some enzymatically inactive sPLA2 homologues (with Lys49 in active site) from *Bothrops* snake venoms display presynaptic neurotoxicity on nerve-muscle preparations due to the general membrane-destabilizing effect of these toxins without phospholipolysis [48]. Thus, Lys49 sPLA2 homologue bothropstoxin-I from *Bothrops jararacussu* snake venom produced the irreversible neuromuscular blockade of mouse phrenic nerve-diaphragm preparations and this blockade was prevented by manganese solution (0.9 mM) [49]. The authors hypothesized that bothropstoxin-I may act through calcium channels which are protected by manganese from the toxin effect. It should be mentioned that no signs of neurotoxicity were observed after bothropic envenomations. This discrepancy may be explained by species specificity of the *Bothrops* venoms. In vitro neurotoxicity studies were carried out on mouse preparations. Another possibility is that the amount of venom injected at *Bothrops* bite is not sufficient to produce neurotoxic signs in humans. This contradiction was discussed in details in the review [48]. 

Several presynaptic neurotoxins have been studied by mutagenesis, crystallography, and by biophysical methods to identify regions and particular residues within a molecule responsible for presynaptic neurotoxicity and other activities. Among the known monomeric presynaptic sPLA2 of group IIA, which X-ray structures are obtained to date, are ammodytoxin A and ammodytoxin C from the venom *V. ammodytes ammodytes* (a true viper) [40] as well as β-agkistrodotoxin from *Agkistrodon halys pallas* [50]. Ammodytoxin A (a strong presynaptic neurotoxin) differs from ammodytoxin C (a strong anticoagulant) by only two amino-acid substitutions (Phe124 > Ile and Lys128 > Glu), suggesting these residues are involved in toxicity and anticoagulant activity. The residues identified by mutagenesis as being important for presynaptic neurotoxicity are located in the C-terminal region of ammodytoxin A (Tyr 115, Ile116, Arg118, Asn119, Phe124), in the N-terminal helix A (Met7 and Gly11), and in the vicinity of Phe24 [40,51] (standard amino acid numbering according to [52]). Trimucrotoxin from *Protobothrops mucrosquamatus* venom is a representative of monomeric presynaptic neurotoxic Asn6-sPLA2s that are found exclusively in pit vipers. Its neurotoxic sites proven by mutagenesis include residues Asn1, Asn6, Lys7, Ile11, Met12, Gly53, Thr79, His108 and Met118 [53]. So, these data suggest that the sites responsible for presynaptic neurotoxicity in sPLA2s are located in the N-terminal helix A and in C-terminal region.

In β-agkistrodotoxin molecule, two spatially adjacent regions, turn 55–61 and stretch 85–91, are remarkably different from those of non-neurotoxic sPLA2s and likely involved in the recognition of a specific receptor at the presynaptic membrane. The structural comparison of the potential recognition site with non-neurotoxic isoforms shows reduced hydrophobicity and the absence of residues with bulky hydrophobic side chains (e.g., Trp) that serve as membrane anchors. This structural characteristic of β-agkistrodotoxin may explain the reduced non-specific binding of the toxin to the non-target membrane before it finds the presynaptic membrane and binds to the putative receptor [50]. 

Dimeric presynaptic neurotoxins are more represented and include, in particular, crotoxin and its analogues from *Crotalus* pit vipers, viperotoxin F from *D. russelii* as well as sPLA2s HDP-1 and HDP-2 from *V. nikolskii*. To date, X-ray structures are obtained for several dimeric neurotoxins including isoform CA_2_CBb of crotoxin from *C. durissus terrificus* venom, as well as tetrameric complex formed by two dimers of crotoxin CB isoforms CBa_2,_ CBc and CBd [40,54]. As discussed earlier, CA subunit increases the lethal potency of the basic subunit CB by directing the heterodimeric crotoxin to a specific membrane receptor. Thus, residues ensuring CA-CB interaction are thought as essential for presynaptic neurotoxicity. The residues Trp31 and Trp70 of the CB subunit (isoform CBb) make non-covalent interactions with the Asp99 and Asp89, respectively, of the β-chain of CA_2_. Another residue important for presynaptic neurotoxicity is assumed Trp80. The C-terminal region of CB does not participate in the formation of the crotoxin complex, but might be involved in the neurotoxic activity of crotoxin. This region in CBd, CBc and CBb isoforms is strongly positively charged. The presence of two negatively charged residues: Glu116 and Glu128 in CBa2 (instead of Gly116 and 128) results in a significant change in surface charge of the molecule, which may affect the binding to specific receptors and the overall toxicity of the crotoxin [40,54].

Presynaptic neurotoxins may penetrate the cell membrane and disturb the mediator release not only at neuromuscular junction. At least crotoxin CB subunit can be internalized in cerebrocortical synaptosomes, independently of the presence of CA or of own catalytic activity. Therein, it induces calcium-dependent glutamate release via N and P/Q calcium channels [55]. Many details of this internalization have not been determined to date. This activity may explain why the intraperitoneal injection of crotoxin induces behavioral changes in rats by increasing their emotional states and decreasing their exploratory behavior [56]. β-Agkistrodotoxin inhibits the K^+^-evoked Ca^2+^-dependent release of transmitters (glutamate, aspartate, glycine and GABA) from cerebrocortical synaptosomes in a concentration-dependent manner. However, it practically does not affect ionomycin-stimulated Ca^2+^-dependent transmitter release; this means that the toxin blocks transmitter release by decreasing the entry of Ca^2+^ into cytoplasm. Thus, β-agkistrodotoxin binding to a high affinity site in synaptic membrane may be coupled to the release of different transmitters [57].

### 3.2. Postsynaptic Neurotoxicity

Postsynaptic neurotoxins include elapid α-neurotoxins known to block postsynaptic nicotinic acetylcholine receptors (nAChRs). The first viperid neurotoxic sPLA2 referred to as postsynaptic neurotoxin was dimeric vipoxin from *V. ammodytes ammodytes* venom (cited in [58]); however, the data about the postsynaptic activity of this toxin are vague. Bitanarin from the puff adder venom *B. arietans* is the sPLA2 with well-established postsynaptic activity. Bitanarin competes with [¹²⁵I]iodinated α-bungarotoxin (αBgt), a typical α-neurotoxin, for binding to human α7 and *Torpedo californica* nAChRs, as well as to acetylcholine-binding protein from *Lymnaea stagnalis*, the IC_50_ values being 20 ± 1.5, 4.3 ± 0.2, and 10.6 ± 0.6 μM, respectively. It also blocks reversibly acetylcholine-elicited current in isolated *L. stagnalis* neurons with IC_50_ of 11.4 μM [59]. The toxin has molecular mass of 27.4 kDa; its 28 cysteine residues form 14 disulphide bonds within a single polypeptide chain, N-terminal sequence of which shows high similarity to that of PLA_2_s from snake venoms. Indeed, bitanarin possesses high phospholipolytic activity of 1.95 mmol/min/μmol. 

Postsynaptic blockage appears to be a property fairly common among viper sPLA2s. Blocking effect on isolated *L. stagnalis* neurons was shown also by two dimeric sPLA2s (IIA group) from *V. nikolskii* venom and three monomeric sPLA2s from *V. ursinii* venom: vurtoxin, Vur-PL and enzymatically inactive Vur-S49, all from IIA group. Vur-S49 is a moderate nAChR antagonist on isolated *L. stagnalis* neurons possessing IC_50_ of 2.18 µM while vurtoxin, Vur-PL and sPLA2s from *V. nikolskii* are weaker. The affinities of sPLA2s for nAChRs and AChBPs have been evaluated by radioligand assay in competition with [^125^I]-labeled αBgt for binding to human α7 and *T. californica* nAChRs. Vurtoxin and Vur-PL show a similar inhibition of α-Bgt binding to α7 nAChR, IC_50_ values being 14 ± 5 µM and 29 ± 2 µM, respectively; at the same time, vurtoxin is the most effective inhibitor of α-Bgt binding to *T. californica* nAChR with IC_50_ of 260 ± 20 nM while Vur-PL2 is practically inactive. Authors believe that this inhibition is not the result of phospholipolytic activity because: (i) blockade is reversible (though slowly); (ii) Vur-S49 possessing no phospholipolytic activity suppresses the agonist-evoked current and its inhibitory potency is higher than that of structurally similar but enzymatically active vurtoxin (2.18 versus 10.5 µM); and (iii) experiments on *L. stagnalis* neurons have been conducted in Ca^2+^-free (Ba^2+^ substituted) medium [60] which abolishes phospholipolytic activity. Crotoxin completely inhibits [^125^I]-α-Bgt binding to all three targets. The experimental points for crotoxin binding to *Torpedo* and α7 nAChRs are best fit by a two-site model, with the difference in affinities between the two sites being about an order of magnitude for *Torpedo* nAChRs (30 and 260 nM) and more than three orders of magnitude for α7 nAChRs (4.9 nM and 15 μM). For both receptors, the high affinity binding sites account for 20–30% of all binding sites [61].

A question arises: do sPLA2s possess pre-, or postsynaptic neurotoxicity, or both? Three sPLA2s, VRV-PL-IIIc, VRV-PL-VII and VRV-PL-IX with molecular weights of 13.003, 13.100 and 12.531 kDa, respectively, have been isolated from the *D. russelii* snake venom and shown to possess phospholipolytic activity. Electrophysiological studies using cultured hippocampal neurons demonstrate that VRV-PL-V and VRV-PL-VII act as both pre- and postsynaptic toxins, while VRV-PL-IX acts as presynaptic toxin [62].

Another question: is an enzymatically-inactive acidic subunit of IIA group sPLA2s involved in its pre- and postsynaptic neurotoxicity? It has been shown that CA subunit is involved in CB protection against non-specific binding and in its direction to specific targets at the presynaptic membrane [20,42,54]. Crotoxin, as well as CB alone, has biphasic effects on nerve-evoked transmitter exocytosis characterized by a transient initial facilitation followed by a sustained decay. Crotoxin and CB reduce nerve-evoked acetylcholine release by 60% and 69%, respectively, but only the heterodimeric crotoxin decreases the amplitude of nerve-evoked muscle twitches. This shows that crotoxin exerts a presynaptic inhibitory action on acetylcholine release that is highly dependent on its intrinsic phospholipolytic activity. At the same time, presynaptic block caused by the toxin is not enough to produce muscle paralysis unless a concurrent postsynaptic inhibitory action is also exerted by the crotoxin heterodimer CB-CA [63]. 

**Figure 1:**
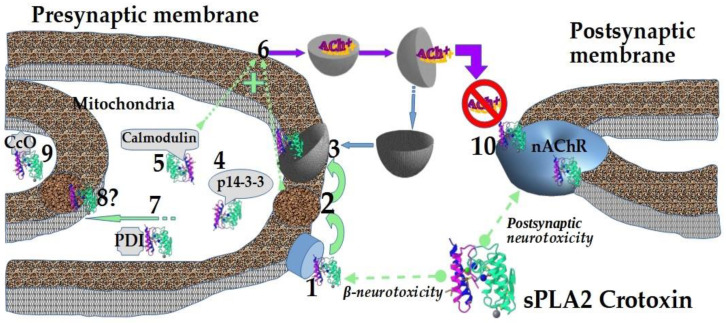
The main processes within neuro-muscular junction known to be affected by snake venom sPLA2 crotoxin: 1—binding to a specific site at presynaptic membrane (presumably to N-type sPLA2 receptor); 2—a general membrane-destabilizing effect and phospholipolysis; 3—translocation across the membrane using the synaptic vesicle recycling machinery; 4—binding to proteins 14-3-3 for localization inside the nerve ending; 5—stabilization by calmodulin with increasing the enzymatic activity; 6—enhancement of exocytosis of acetylcholine (Ach) by products of phospholipolysis; 7—retrograde trafficking provided by protein disulfide isomerase (PDI); 8—putative crossing the outer mitochondrial membrane; 9—binding to subunit II of mitochondrial cytochrome c oxidase (CcO); 10—binding to and block of a nicotinic acetylcholine receptor (nAChR) at postsynaptic membrane. Crotoxin structure is from PDB bank (ID 3R0L).

Thus, the effects of sPLA2s on the nervous system are multidirectional and involve interactions with different proteins. These interactions are schematically presented on Figure 1 taking crotoxin as an example.

### 3.3. sPLA2s in Pain and Analgesia

sPLA2s exhibit various biological activities and, due to their phospholipolytic activity leading to the formation of precursors of various mediators, are involved in many pathophysiological processes, including inflammation and pain. sPLA2-induced hyperalgesia is mediated through pro-inflammatory stimuli: extracellular PLA2-derived pro-inflammatory mediators, including prostaglandins, prostacyclins, thromboxanes, leukotrienes, and others [64]. 

Crotoxin is a unique snake venom sPLA2 for which anti-nociceptive activity has been studied in detail (see review [65]). It has been established that the opioid system seems not to be involved in this activity; the adrenergic one might be employed partly and data on participation of muscarinic receptors are inconsistent. Briefly, in mice and rats, crotoxin at peripheral (intraperitoneal) or central (intracerebral ventricular or periaqueductal gray area) injections induces antinociception. Neither muscarinic nor opioid receptor inhibition interfere with the antinociceptive effect [66]. Crotoxin solution prevents the development of neuropathic pain being applied immediately (0.01 mM for 10 s) after rat sciatic nerve transection. In contrast to the previously cited work, this long-lasting antinociceptive effect (persists for 64 days after the local application) involves central muscarinic receptors and is partially mediated by the activation of α-adrenoceptors and 5-HT receptors [67]. An intracerebroventricular injection of crotoxin inhibits pain-evoked electrical discharge of thalamic parafascicular nucleus neurons, an important relay in the ascending nociceptive pathways. This effect is not influenced by muscarinic and opioid receptor antagonists [68]. Blood oxygen level dependent functional Magnetic Resonance Imaging analysis has confirmed the participation of specific areas of the CNS in the antinociceptive effect of crotoxin, not only in brain input structures, but also in higher order processing structures, such as primary and secondary somatosensory cortices, which are relevant for pain perception [69]. When conjugated with SBA-15 (a drug delivery system) crotoxin induces a long-lasting reduction of mechanical hypernociception, without modifying the previously known pathways involved in antinociception. Atropine (a muscarinic antagonist) completely reverses the antinociceptive effect of the conjugate while yohimbine (an α2-adrenergic antagonist) makes it only partially [70]. Presumably, the exceptional antinociceptive effect of crotoxin among all sPLA2s may be related with the unique structure of its acidic subunit CA [40,71]. Non-neurotoxic bee venom sPLA2 (group III) prevents oxaliplatin-induced neuropathic pain, however, it does not contain CA-like subunit; unlike crotoxin, its anti-nociceptive effect is due to suppressing immune response, but not to a direct neurotoxic activity [72]. 

An anti-nociceptive effect observed in vivo should be considered, taking into account the general toxicity inherent in presynaptic neurotoxins. So, neurotoxic sPLA2 HDP-2 from *V. nikolskii* venom increases the hot plate latencies, but this effect is only observed at the maximal tolerated dose. Given that at this dose the animals manifest symptoms of intoxication, including depression and a marked decrease in locomotor activity, the observed effect in the hot plate test may be the result of a general intoxication rather than a specific decrease in pain sensitivity [71].

It should be noted that some viperid venoms contain myotoxins, which are sPLA2s lacking enzymatic activity due to the substitution of Asp49 in active site by Lys, Ser or Arg. They cannot produce the mediator precursors mentioned earlier, but they induce muscle damage and pain. The latter effect obviously affects the nervous system, but the exact target has not yet been established. A myotoxin, non-enzymatic Lys49-PLA2 from *Bothrops* venoms, induces ATP and K^+^ release from muscle cells that can directly induce pain by activating purinergic receptors or inducing the membrane depolarization of the peripheral sensory nerve [73]. BomoTx, a myotoxin-like monomeric Lys49-sPLA2 from *B. moojeni* (Brazilian lancehead pit viper), excites a cohort of sensory neurons from rat trigeminal ganglia which consist of both nonpeptidergic C fibers and peptidergic Aδ fibers through a mechanism involving the ATP release and activation of P_2_X_2_ and P_2_X_3_ purinergic receptors [74]. BomoTx is not a direct purinergic receptor agonist or modulator; it evokes intracellular calcium release from a specific population of sensory neurons that leads to hemichannel pannexin opening and release of ATP to the extracellular environment. The activation of transient responses in specific sensory neurons that express purinergic receptors P_2_X_2_ and/or P_2_X_3_ elicits acute pain behaviors and heat hypersensitivity [74]. 

## 4. Three-Finger Toxins

The three-finger toxins (TFTs) are abundant in neurotoxic snake venoms, typically representing the main pool of components of elapid venoms. TFTs adopt their name according to their characteristic spatial structure: three β-sheet loops (fingers) protrude from the central core, stabilized by four conserved disulfide bonds. They belong to the Ly6/uPAR superfamily of proteins, which modulate inter alia and the nAChR functions in mammalian CNS. The TFTs contain from 57 to 82 amino acid residues, with some toxin types having an extra fifth disulfide bond, located in either their central loop II (“long-chain” neurotoxins) or N-terminal loop I (“non-conventional” neurotoxins). The position of this bond affects the toxin biological activity which is indeed very diverse in TFTs [75].

Earlier, it was generally believed that viperid venoms could not contain TFTs. Over time, this turned out not to be true. 

In 1987 there was the first indication published that viperid venom might contain TFTs. α-Agkistrodotoxin, a putative α-neurotoxin with a molecular weight of about 8 kDa, was isolated from the venom of the pit viper *A. halys* [76]. Its amino acid sequence and putative relation to TFTs remained unknown; nevertheless, the toxin inhibited the binding of radioactive αBgt to nAChR of cultured myotubes (IC_50_ = 2 nM) and the carbachol-induced influx of cations through the nAChR (IC_50_ = 60 nM); it also cross-reacted with antiserum against αBgt [76]. 

Two postsynaptic neurotoxins with similar properties were isolated from the venom of the viper *D. russelli* with a distance of 11 years. The first one, named DNTx I, acted on frog nerve−muscle preparation at a concentration of 4 µg/mL inhibiting the twitch almost completely [77]. Its molecular mass was 6675 Da and N-terminal amino acid sequence was LECNKLQPIASK, being 80% identical to that of a cytotoxin/cardiotoxin from cobra *N. naja atra*. The venom sample used for toxin isolation was from Hindustan Park, Calcutta, India [77]. The second one, DNTx-III, displayed the concentration-dependent inhibition of indirectly stimulated twitches of *Rana hexadactyla* sciatic nerve gastrocnemius muscle preparations [78]. At a concentration of 10 µg/mL, DNTx-III completely inhibited indirectly stimulated twitch response in 30 min. Its molecular mass was 6849 Da and N-terminal amino acid sequence was IRCFITPDUTSQACP, showing a high degree of similarity to cobra α-neurotoxins. The venom sample used in this work was from Hindustan Snake Park, Burdwan, India [78]. Interestingly, despite the extreme dissimilarity of N-terminal sequences, these two toxins show similar biological activity.

More recently, with the proteomic and transcriptomic analysis of venoms and venom glands, the presence of TFTs in viperids was undoubtedly shown. Thus, transcripts encoding TFTs have been detected in venom gland transcriptomes of several Viperidae species, including *Lachesis muta* [79] and *C. atrox* [80]. Genomic DNA encoding TFT was reported for *Sistrurus catenatus* [81]. By a proteomic approach, the presence of TFTs was discovered in several viperid venom, including *Azemiops feae* [82], *E. ocellatus* and *B. arietans* [16]. However, the functional activity of proteins discovered in these ways remains unknown. To address the issue of biological activity of Viperidae TFTs, two toxins, TFT-AF and TFT-VN, have been obtained by heterologous expression in *Escherichia coli*. Their amino acid sequences were deduced from the nucleotide sequences of cDNA encoding these TFTs in the venom glands of vipers *A. feae* and *V. nikolskii* [83]. These both TFTs belong to the group of non-conventional toxins with the fifth disulfide bonds within the N-terminal loop. The biological activity of the toxins has been studied by electrophysiological techniques, calcium imaging, and radioligand analysis. Both toxins inhibited neuronal α3-containing and muscle-type nAChRs in the micromolar concentration range, but showed very weak antagonism on neuronal α7 nAChR. Thus, viper TFTs function as antagonists of neuronal and muscle-type nAChRs [83] and are true neurotoxins.

A lot of TFTs has been discovered in venom of rear-fanged snakes [84]. All these TFTs are so-called non-conventional toxins, characterized by an additional fifth disulfide bond in the first loop.

Although some toxins, which were able to inhibit [^3^H]α-Bgt binding to nAChR, were found in Duvernoy’s secretions of rear-fanged snakes back in 1999 (in two cat-eyed snakes spp., Colubridae [85]), the first well characterized TFT from the venom of rear-fanged snake *Coelognathus radiatus* (formerly *Elaphe radiatus*, Colubridae) α-colubritoxin was reported in 2003. This toxin was homologous to elapid TFTs, at 1 μM, it blocked twitches of the isolated chick biventer cervicis muscle and significantly inhibited contractile responses to the exogenous nicotinic agonists, acetylcholine and carbachol, but not to KCl, i.e., it was an α-neurotoxin [86]. At that time, it was amazing; to date it has been established that TFTs dominate in the venom of several rear-fanged snake genera, including cat-eyed snakes [9]. Sometimes, these TFTs are N-terminally blocked by a pyroglutamic acid residue [9,86]. This was an obstacle which did not allow Broaders et al. [85] to establish the affiliation of the found toxins with the TFT in 1999. 

Besides α-colubritoxin, there is a report on the TFT presence in rear-fanged snake venoms, which are predominantly hemotoxic; the proteomic approach has revealed TFT in the venoms of Asian green vine snake (*Ahaetulla prasina*) and Puerto Rican racer (*Borikenophis portoricensis*) [87].

## 5. Other Components of Viperid Venoms with Postsynaptic Neurotoxicity

### 5.1. Waglerins

**Figure 2:**
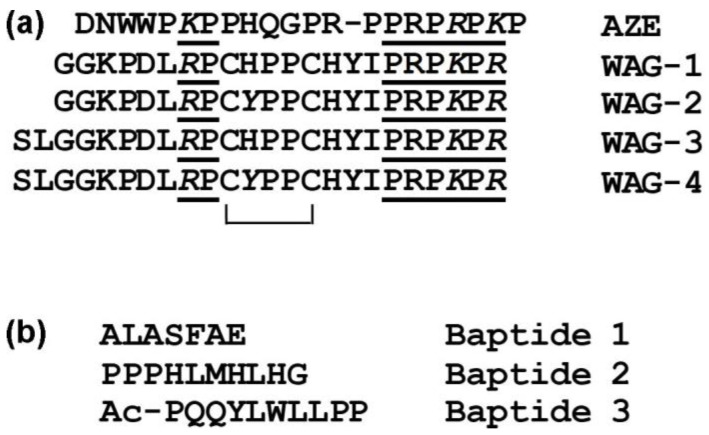
(**a**) Amino-acid sequences of azemiopsin and waglerins. Homologous fragments are underlined. AZE—azemiopsin (UniProt KB-B3EWH2) from *A. feae*; WAG-1 (UniProt KB-P24335), WAG-2 (UniProt KB-P58930), WAG-3 (UniProt KB-P24335) and WAG-4 (UniProt KB-P58930) are waglerin-1, 2, 3 and 3, respectively, from *T. wagleri*. (**b**) Amino acid sequences of baptides from *B. arietans*.

Waglerins are highly basic (pIs of 9.6–9.9) short proline-rich peptides (seven Pro residues per 22 or 24 total amino acids) with one intramolecular disulfide bond (Figure 2a). Four individual waglerins highly homologous to each other were isolated from pit viper *Tropidolaemus wagleri* (formerly *Trimeresurus wagleri*) venom [88,89]. An analysis of transcriptome of *T. subannulatus* pit viper venom gland revealed the nucleotide sequences encoding precursor of the C-type natriuretic peptide, the pre-pro region of which contained a sequence with high degree of identity to waglerins [90].

Waglerins are lethal peptides with an LD_50_ of 0.22–0.58 mg/kg i.p. in adult mice. Neonatal mice are resistant to the lethal effect of waglerin-1. Waglerin-1 at 4 μM reversibly blocks the indirect twitch of the mouse phrenic nerve-hemidiaphragm preparation, while at concentrations up to 40 μM it does not block the indirect twitch of the rat diaphragm. Mice are paralyzed by a 0.5 μg/g intravenous injection during as soon as 5 min, while rats are completely resistant to dose 10 μg/g [91]. This difference is explained by different affinities of waglerin to mouse and rat nAChRs. It interacts 100-fold weaker with rat nAChR than with mouse one [92]. Moreover, it has been found that waglerin distinguishes two agonist/antagonist binding sites in nAChRs. Its interaction with mouse nAChR demonstrates two distinct dissociation constants differing by 2100-fold, with more tight binding to the α-ε than to the α-δ site [92]. This difference is 80 for the rat receptor. At human nAChR, waglerin shows 70-fold lower affinity at the α-ε site and 10-fold lower affinity at the α-δ site [92]. The use of knockout mice lacking the gene coding for ε-subunit have also shown that waglerin-1 selectively blocks the mouse muscle nAChR containing the ε-subunit [93].

There is an indication that the inhibiting action of waglerins at a nerve-muscle junction may be not only post-synaptic. So, sub-micromolar waglerin concentrations depresses endplate currents (EPCs) produced in response to nerve stimulation. Since quantal content of EPCs is not altered, it appears that the site of action is post-synaptic. However, higher concentrations (1.4–2.9 μM) also inhibit spontaneous release of transmitter. Nerve stimulation in the presence of waglerin-1 causes ‘rundown’ of EPC amplitude, demonstrating that the toxin acts presynaptically to impede transmitter release [94].

Waglerins are not so specific to nAChR. Thus, waglerin-1 in co-application with γ-aminobutyric acid (GABA) increases whole cell current response to GABA (I_GABA_) for 54% isolated murine hypothalamic neurons and suppresses I_GABA_ for 30% neurons examined, with IC_50_ 24 μM for both effects. This difference may be explained by variation in the subunit composition of murine GABA_A_ receptors. The potentiating effect of waglerin-1 on I_GABA_ mimics diazepam effect suggesting action at the benzodiazepine site on certain type of GABA_A_ receptor with increase its affinity for agonist. In contrast, the depressant effect of waglerin-1 shows a positive correlation with a similar action of Zn^2+^ suggesting a competitive inhibition of another type of GABA_A_ receptor [95]. Waglerin-1 depresses I_GABA_ when co-applied with GABA also for nucleus accumbens neurons of 3- to 7 days old rats. This effect of waglerin-1 is competitive and voltage dependent, with IC_50_ of 2.4 μM that 10-fold lower than that for hypothalamic neurons. The effect is significantly reduced for neurons dialyzed with H-89 (PKA inhibitor) but not chelerythrine (PKC inhibitor) suggesting that phosphorylation state modulates GABA_A_ receptor sensitivity to the inhibitory effect of waglerin-1 [96]. 

Interestingly, on the basis of waglerin, a tripeptide synthetic compound dipeptide diaminobutyroyl benzylamide diacetate was designed which was used in a skin cream with the trade name Syn^®^-Ake [90]. It relaxes the facial muscles and such way reduces wrinkles.

### 5.2. Azemiopsin

Azemiopsin, from the *A. feae* viper venom (sub-family Azemiopinae within Viperidae), is a basic polypeptide of 21 residues and, unlike the overwhelming majority of snake toxins, does not contain cysteine residues (Figure 2a). Nevertheless, according to circular dichroism measurements, this peptide adopts a β-structure. 

Azemiopsin efficiently competes with α-Bgt for binding to *Torpedo* nAChR (IC_50_ 0.18 ± 0.03 μM) and with lower efficiency to human α7 nAChR (IC_50_ 22 ± 2 μM) [97]. It dose-dependently blocks acetylcholine-induced currents in *Xenopus* oocytes heterologously, expressing human muscle-type nAChR, and is more potent against the adult form (α1β1εδ, EC_50_ 0.44 ± 0.1 μM) than the fetal form (α1β1γδ EC_50_ 1.56 ± 0.37 μM). The peptide has no effect on GABA_A_ (α1β3γ2 or α2β3γ2) receptors at a concentration up to 100 μM or on 5-HT(3) receptors at a concentration up to 10 μM [97].

Azemiopsin resembles waglerins in that it blocks muscle-type nAChR preferring its adult form with similar affinity, shares a homologous C-terminal hexapeptide, and seems to specifically evolve at the same site as a part of the pre-pro region of a C-type natriuretic peptide as a putative waglerin from *T. subannulatus* [90]. The relationship of azemiopsin with waglerins suggests that the two types of nAChR antagonists share a common molecular evolutionary history [90]. However, azemiopsin unlike waglerins is able to block α7 nAChR but unable block GABA_A_ receptors and it is the first natural peptide toxin that blocks nAChRs and does not possess disulfide bridges.

The LD_50_ values for azemiopsin in mice are 2.57 ± 0.27 mg/kg i.p., 0.51 ± 0.06 mg/kg i.v., and 0.732 ± 0.13 mg/kg i.m. At a dose of 0.3 mg/kg and lower (single i.m. injection in mice) and at a dose 0.1 mg/kg (i.m. daily during 14 days in rats) it almost does not display any adverse effects [98]. Giving specific inhibiting activity against muscle nAChR and relative low toxicity, it may be considered as a good candidate for local non-depolarizing muscle relaxant. Due to its highly basic nature, azemiopsin is easily incorporated in nanomaterials based on sulfated polysaccharides [99]; this can prolong its presence in the bloodstream.

### 5.3. Baptides

Three peptides called baptides 1, 2 and 3 have been purified from puff adder *B. arietans* venom. Baptide 1 comprises seven amino acid residues, while baptides 2 and 3–10 residues, the latter being acetylated at the N-terminus (Figure 2b). They do not contain cysteine residues. Baptide 3 and 2 block acetylcholine-elicited currents in isolated *L. stagnalis* neurons with IC_50_ of about 50 μM and 250 μM, respectively. Baptide 2 blocks acetylcholine-induced currents in muscle nAChR heterologously expressed in *Xenopus* oocytes with IC_50_ of about 3 μM. The peptides do not compete with radioactive α-Bgt for binding to *Torpedo* and α7 nAChRs at concentration up to 200 μM that suggests non-competitive mode of inhibition. Calcium imaging studies on α7 and muscle nAChRs heterologously expressed in Neuro2a cells show that baptide 2 inhibits acetylcholine-induced calcium current through α7 receptor with IC_50_ of 20.6 ± 3.93 μM. The suppression of maximal response to acetylcholine by about 50% on both α7 and muscle nAChRs is observed at baptide 2 concentration of 25 μM, the value being close to IC_50_ on α7 nAChR [100]. 

### 5.4. C-Type Lectin-like Proteins (CTLPs)

The reduction of animal locomotor activity and exploration under influence of CTLPs from viperid venom has been reported [22] and explained by hemotoxic effects of these compounds. Unlike that, CTLPs called emunarecins EM1 and EM2 from the saw-scaled viper *E. multisquamatus* inhibit fluorescent α-Bgt binding to both muscle-type nAChRs from *T. californica* and human neuronal α7 nAChRs. EM1 at 23µM and EM2 at 9µM almost completely prevent fluorescent α-Bgt binding to muscle-type nAChR. Interaction with human neuronal α7 nAChR is weaker; EM1 at the concentration of 23µM blocks the α-Bgt binding only by about 40% and EM2 at 9µM by about 20% [101]. These data suggest postsynaptic activity for CTLPs.

## 6. Blockers of Voltage-Dependent Ion Channels

### 6.1. Cysteine-Rich Secretory Proteins

Cysteine-rich secretory proteins (CRiSP) constitute a large family of vertebrate proteins responsible for various biological activities, including the reproduction and blockage of ion channels. Snake venom CRiSPs is a toxin family with a high degree of sequence similarity. They comprise a single polypeptide chain of 20–30 kDa and 16 highly conserved cysteine residues that form eight disulfide bonds, with 10 of these residues residing in the cysteine-rich domain at the C-terminus [102]. CRiSPs lack proteolytic and any enzymatic, hemorrhage and coagulant activity [103]. These proteins widely spread thorough snake venoms, and, in some venoms, they are present in considerable amounts. Nevertheless, their biological significance still has not been completely established. Most of the snake venom CRiSPs whose functions are known show effects on smooth muscle contraction, affecting, as a rule, various ion channels. So, BaltCRP, a CRiSP from *B. alternatus* venom with a molecular mass of 24.4 kDa and an isoelectric point of 7.8, at 1 μM, inhibits approximately 26% of K_v_1.1, 14% of K_v_1.3, 17% of K_v_2.1 and 21% of Shaker potassium currents and has no effect on K_v_1.2, K_v_1.4, K_v_1.5 and K_v_10.1 [104]. Helicopsin, a CRiSP of ~20 kDa from the rear fang snake *Helicops angulatus*, has robust neurotoxic activity, causing respiratory paralysis in mice at 16 μg/mouse and possessing LD_50_ of 5.3 mg/kg i.p. [105]. Helicopsin is classified as neurotoxin basing only on neurological symptoms typical for depolarizing neuromuscular blocking neurotoxins, including edginess, ataxia, convulsions, flaccid paralysis of respiratory muscles, and death, which occurs from 6 to 8 min, but appropriate research in vitro has not been carried out [105]. 

Ablomin from the venom of the Japanese Mamushi snake (*A. blomhoffi*) inhibits contraction of rat tail arterial smooth muscle elicited by high K^+^-induced depolarization in the 0.1–1 micromolar range (about to 45–75% from control), but does not block caffeine-stimulated contraction. The effective concentration for smooth muscle contraction force herein is in almost the same range (K_i_ of 0.21 µM for ablomin) as that of L-type Ca^2+^ channel blocker calciseptine from mamba venom (Elapidae). Calciseptine demonstrates IC_50_ of 0.23 µM on rat aorta depolarization-induced contraction. However, the blockage by ablomin is not complete even at the concentration of 1 µM; higher concentrations of ablomin (3 µM) do not induce further inhibition, but rather reduces the extent of inhibition. Two more CRiSPs that are homologous to ablomin have been isolated and the corresponding cDNAs encoding these proteins have been cloned. One of these homologous proteins, triflin from the pit viper venom *Trimeresurus flavoviridis* (formerly *P. flavoviridis*), also inhibits a high K^+^-induced contraction of the artery, the effect being comparable to that of ablomin [106]. Another homologue, tigrin from tiger keelback venom *Rhabdophis tigrinus tigrinus* (subfamily Natricinae within Colubridae) seems to lack such neurotoxicity [106].

In addition, other CRiSPs, including piscivorin from *A. piscivorus piscivorus* venom as well as catrin-2 and catrin-1 from *C. atrox* venom, have also been reported to inhibit arterial smooth muscle contraction evoked by high K^+^ to 79.2 ± 4.5%, 70.7 ± 3.4%, and 90.3 ± 4.1% of control, respectively, at a concentration of 1 μM. None of these proteins inhibits contractions evoked by caffeine [107].

### 6.2. Kunitz-Type Protease Inhibitors

Snake venom Kunitz-type serine proteinase inhibitors (KSPIs) have a structural similarity to aprotinin (bovine KSPI) and consist of approximately 60 amino acids. They have three conserved disulfide bridges responsible for the stability of the molecule and two antiparallel β-strands, which are linked by a β-hairpin in the central part of the molecule. With respect to functional activities, a positive Darwinian selection has divided snake venom KSPIs into two major groups: non-neurotoxic (trypsin and chymotrypsin inhibitors) and neurotoxic (potassium and calcium blockers) snake venom KSPIs [108].

Neurotoxic KSPIs that interfere with neuronal transmission have been detected in venoms of elapid snakes. So, dendrotoxins from mamba venom (neurotoxic Elapid) block voltage-dependent K^+^ channels at the presynaptic site of the neuromuscular junction. KSPIs from viperid are reported to aim mostly at the blood clothing system [109]. However, a non-covalent rusvikunin complex composed of two KSPIs, rusvikunin and rusvikunin-II, from Russell’s viper *D. russelii russelii* venom along with an anticoagulant effect exhibits increased respiration rate, dyspnea, difficulty in movement and hind-limb paresis at a dose of 5 mg/kg in mice prior to death. These symptoms may indicate possible neurotoxic activity [110]. 

KSPI VaaChi from the venom of the nose-horned viper (*V. ammodytes ammodytes*) significantly increases the amplitudes of an indirectly evoked simple muscle contraction of the mouse hemidiaphragm, the end-plate potential and the miniature end-plate potential. What is the mechanism behind facilitation of neuromuscular transmission by VaaChi has not been established yet; however, the blocking of K^+^ channels does not seem to be the most probable option [111].

### 6.3. Crotamine

Crotamine is a 42-amino acid peptide from the venom of *C. durissus terrificus* rattlesnake. It is a member of the small basic myotoxin family, which is distributed through the venoms of *Crotalus* genus and belongs to β-defensin superfamily. Its fold (with triple-stranded β-sheet, N-terminal α-helix, and the pattern of three disulfide bridges) and potential surface resemble the structural features of β-defensin-like peptides. When injected (i.p. or s.c.) at non-toxic doses in mice, crotamine induces a time-dose dependent analgesic effect 30 times more potent than morphine (500-fold by molar ratio), and naloxone antagonizes this an opioid-like activity [112]. However, the antinociceptive effect of recombinant crotamine is not affected by pre-treatment with naloxone [113]. Crotamine induces spastic paralysis in the hind limbs. Earlier, it was considered as an inductor of the depolarization of membrane potential of the skeletal muscle and influx of Na^+^ [114]. This effect was prevented by decreasing extracellular Na^+^ or by tetrodotoxin, albeit the latter and other sodium channel blockers were not able to mimic the hind-limb paralysis produced by crotamine. Moreover, it has been shown [115] that crotamine does not affect directly mammalian voltage-dependent Na^+^ channels. Giving three-dimensional structure resemblance with human antibacterial β-defensins and using computational docking, it has been proposed that crotamine should act as a voltage-dependent potassium channel blocker [116]. Indeed, it inhibits K_V_1.1, K_V_1.2, and K_V_1.3 channels with an IC_50_ of ~200–450 nM—rapidly, reversibly, selectively and voltage-independently without both a shift in the midpoint of activation and change in ion selectivity. At the same time, at 3μM, it has no effect upon other KV channel isoforms from the Shaker (K_V_1.4–K_V_1.6 and Shaker IR), Shab (K_V_2.1), Shaw (K_V_3.1), Shal (K_V_4.3 and K_V_4.3), and erg (K_V_11.1) subfamilies as well as upon sodium channels (Na_V_1.2, Na_V_1.3, Na_V_1.5, and the insect channel DmNa_V_1) [117]. So, the action of crotamine on sodium channels seems to be dependent on experimental condition. It is able to increase the force of contraction of isolated skeletal muscles induced by constant electrical stimuli, with the involvement of both voltage-gated sodium and potassium channels [118]. Anyway, crotamine at low concentrations improves neuromuscular transmission; it (0.25 μg/kg) is more efficient than neostigmine (17 μg/kg) for enhancing muscular performance in experimental myasthenia gravis in rats [119].

Given the overexpression of K_V_1.1 and K_V_1.3 at carcinogenesis and the cell-penetrating ability of the small basic myotoxins, crotamine is now considered as a promising anti-tumor agent. Other possible medicinal applications of this versatile compound are not related to its neurotoxicity [120].

The co-administration of phenotiazine thioridazine (THD), an antipsychotic that blocks the dopamine D2 receptors, speeds up 2-fold the advent of crotamine-evoked hind-limb paralysis. Interestingly, crotamine, in turn, reduces the time for sedation produced by THD (by 25–40%), thus demonstrating possible central action. The pre-incubation of an isolated diaphragm preparation with THD induces a decrease by about 30% of the maximum contraction force produced by 30 nM crotamine, whereas voltage-gated potassium channel blocker 4-aminopyridine enhances the crotamine-induced rise of contraction force by 2-fold [121].

## 7. Sarafotoxins

**Figure 3:**
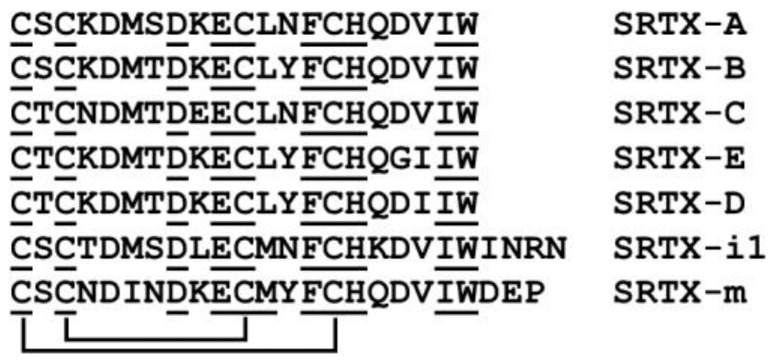
Amino acid sequences of sarafotoxins. Disulfide bonds are shown as lines connecting cysteine residues. Identical amino acid residues are underlined. SRTX-A (UniProtKB-P13208), SRTX-B (P13208), SRTX-C (P13208), SRTX-E (P13208), and SRTX-D (P13211) are sarafotoxins a, b, c, e, and d from *A. engaddensis* venom, respectively. SRTX-i1 (P0DJK0) is sarafotoxin i1 from *A. irregularis* venom; SRTX-m (Q6RY98) is sarafotoxin m from the venom of *A. microlepidota microlepidota*.

Sarafotoxins are peptides comprising 15–30 amino acid residues with two conserved disulfide bridges. Four major isopeptides S6a-d are composed of 21 amino acids (Figure 3). They have been found solely in the venoms of the snake from *Atractaspis* genus (Atractaspididae, Colubroidea). Sarafotoxins share high structural and functional homology with the vertebrate endothelins and interact with endothelin receptors, which can modulate the contraction of cardiac and smooth muscles in different tissues. Sarafotoxins are among the most lethal snake toxins ever described, causing death within minutes in mice with LD_50_ of 0.01–0.3 µg/g and under one hour in humans [122].

Endothelin signaling through endothelin type B receptors (ET_B_R) participates in increasing neuronal activity. Therefore, the activation of ET_B_R on sympathetic nerves would increase blood pressure through an adrenergic-mediated mechanism. To determine the pressor response to the selective ET_B_R agonist sarafotoxin c (S6c), ET_B_R deficient rats, which express functional ET_B_R only on adrenergic neurons, have been used [123]. A dose-dependent pressor response to S6c in ET_B_R-deficient rats is reversed by prazosin, an α1-adrenergic receptor antagonist, and is augmented by propranolol, a β-adrenergic receptor antagonist. Heart rate is mostly unaffected by S6c alone or by S6c with either prazosin or propranolol. These results suggest that ET_B_R activation on sympathetic neurons induces an increase in blood pressure mediated through α1-adrenergic receptor signaling [123].

Most of the works on sarafotoxins were published at the end of the last century. Thus, it was found that ^125^I-sarafotoxin-b recognized sites in rat brain, particularly in the cerebellum (K_D_ = 3.5 nM) and cerebral cortex (K_D_ = 0.3 nM) [124]. Sarafotoxins possess strong cardiotoxic activity resulting in atrio-ventricular block in vertebrates. Impairment of an electrical signal from the heart atria to the ventricles is varied dependently on a species [125]. Sarafotoxin-6B increased [Ca^2+^]i in individual cerebellar astrocytes in cell culture through activation of endothelin receptors. Ca^2+^ influx was predominantly non-voltage-dependent, although some entry through a dihydropyridine-sensitive pathway also appeared to occur. [126]. All of these data suggest the presence of molecular targets in excitable tissues, including neurons, that may be encountered along signal propagation pathways.

Presently, sarafotoxins are used as biochemical tools to study endothelin receptors. So, to clarify the recognition mechanism of the endothelin-like peptide family, a crystal structure of the human ET_B_R in complex with sarafotoxin S6b was determined [127].

The data about neurotoxins discussed in this review are summarized in the table (Table 1). 

## 8. Conclusions and Prospects

Venoms that have a damaging effect on the nervous system or blood are considered as neurotoxic or hemotoxic, respectively. This division is quite conventional. Most venomous snakes belong to the Elapidae or Viperidae families. In general, the elapid venoms comprise toxins affecting the nervous system and are considered as neurotoxic. The action of viperid and most of the rear-fanged snake venoms are directed mainly on blood coagulation and they are regarded as hemotoxic. However, some bites by viperids results in neurotoxic signs [32]. Indeed, different neurotoxins were isolated from hemotoxic venoms. In this review, we have tried to systematize data on such neurotoxins and considered their structural and functional characteristic. First of all, these are phospholipases A2, which manifest pre- or/and postsynaptic neurotoxicity. They are also involved in nociceptive and analgesic effects of venoms as well as in inflammation. A few viperid venoms contain peptide antagonists of nAChRs: waglerins [88,89], azemiopsin [97] and baptides [100]. Some CriSPs ubiquitous practically in all snake venoms including viperids and colubrids interfere with ionic channels functions. Typical TFTs have been discovered in colubrid and viperid venoms and transcripts encoding putative TFTs have been found in transcriptomes of venom glands. Viperid venoms comprise some other toxins manifesting neurotoxicity (crotamine, C-type lectin-like proteins etc.). This indicates the broad distribution of neurotoxins in the venoms earlier considered as hemotoxic. These toxins belong to diverse families ranging from large multi-subunit proteins to short peptide neurotoxins, and the latter ones might avoid neutralization by antivenom. As discussed in this review, venoms of the snakes from the genera *Agkistrodon*, *Bitis*, *Crotalus*, *Daboia*, *Vipera* and some others possess neurotoxicity. It cannot be excluded that viperid snakes from other genera may manifest or acquire neurotoxicity. Thus, the neurotoxic effect of the venoms should be considered in the treatment of bites by venomous snakes from the Viperidae family. 

As discussed above, some toxins exhibit very promising effects, such as analgesic ones. These toxins can serve as the basis for biomedical and clinical research. First of all, mention should be made of crotoxin, whose analgesic activity is well known [66,67,68,69,70]. Interestingly, a phase I clinical trial of the crotoxin was performed on patients with solid tumors and recommended dose for phase II was determined [128]. This trial shows that human patients can be treated with crotoxin and its use as an analgesic may be realized. To prevent its high toxicity and improve safety, nanostructured mesoporous silica (SBA-15) was used as a carrier [70]. The conjugate obtained produced a long-lasting reduction of mechanical hypernociception. Other viperid neurotoxin interfering with pain is crotamine. It produces analgesic effect more potent than morphine (500-fold by molar ratio) [112]. Recent studies have shown that crotamine induces antinociception being orally administered in mice [129]. When solving the problem of toxicity, this result opens up prospects for the further use of crotamine as an analgesic drug.

Ctotoxin and crotamine are small proteinaceous toxins and have the inherent disadvantages of proteins, including the complexity of production, immunogenicity, the lack of stability in the bloodstream, toxicity, etc., which must be overcome before their introduction into practice. Peptide toxins are devoid of a number of these shortcomings. It was already mentioned earlier that an anti-wrinkle cream used in cosmetics was developed on the basis of the peptide neurotoxin waglerin [90]. Preclinical studies carried out for the other peptide neurotoxin azemiopsin showed good prospects for the development of muscle relaxant on the basis of this peptide [98]. Along this line, a patent was issued for the use of azemiopsin in the treatment of muscular dystonia [130].

Although many neurotoxins have already been discovered in hemotoxic venoms, not all such venoms have been fully explored and the discovery of new toxins with unusual properties can be expected. Thus, as a rule, in viperid venoms, TFTs are either present in very small quantities or completely absent despite the fact that mRNA or gene sequences encoding these toxins are found. Colubrid TFTs have been characterized only from small part of venoms including cat-eyed snakes and some others described in this review. It would quite interesting to produce neurotoxins from viperid venom and study their differences from canonical three-finger neurotoxins. Acetylcholinesterase (AChE) has been found in small amounts in several snake venoms including viperid [131]. The physiological role of endogenous AChE is hydrolysis of acetylcholine within a synaptic cleft. Hypothetically, venom AChE may interfere with cholinergic signaling; however, to our knowledge, such an activity has not been reported for AChE from the venom of viperid or colubrid. TRPV1 channels expressed on somatosensory nerve terminals are known to be related with pain response. Some heat-stable structurally-unidentified proteins from the venom of saw-scaled viper *E. coloratus* activate TRPV1 to produce a channel-dependent increase in intracellular calcium and outwardly rectifying currents in neurons and heterologous systems. Interestingly, the channel activation is not mediated by any of its known toxin binding sites. Moreover, although neurotropic activity of nerve growth factor (NGF) has been detected in this venom, TRPV1 activation appears to be independent of NGF receptors [132]. Hemotoxic components predominate in the venom of European rear-fanged snake, *Malpolon monspessulanus monspessulanus*, at the same time, it evokes neurological symptoms. A recent proteomic assay shows no TFTs and only 14% PLA2s in the venom [133]. It would be exciting to establish what compound is responsible for the neurotoxicity. 

Thus, there are broad prospects for further research into neurotoxins in hemotoxic venoms.

## Figures and Tables

**Table 1:** Neurotoxins identified in hemotoxic venoms.

Name	Species (Common Name)	Biological Activity(es)	Protein Family	Structural Type	Size, mol. Weight	Subunit Composition	Other Structural Features	Ref.
Ablomin	*A. blomhoffi* (Japanese Mamushi snake)	K^+^-blocker	CRiSP		221 aa, 24.9 kDa	monomer		[106]
α-Agkistrodotoxin	*A. halys pallas* (Pallas’s pit viper)	Postsynaptic neurotoxin	putative TFT	Putative long-chain α-neurotoxin	8.0 kDa	monomer		[76]
β-Agkistrodotoxin	*A. halys pallas* (Pallas’s pit viper)	β-neurotoxin	sPLA2	Group IIA sPLA2		monomer		[50]
Ammodytoxin A	*V. ammodytes ammodytes* (nose-horned viper)	β-neurotoxin, phospholipolysis, anticoagulant,	sPLA2	Group IIA sPLA2	122 aa, 13.8 kDa	monomer		[40,47,51]
Ammodytoxin C	*V. ammodytes ammodytes* (nose-horned viper)	Anticoagulant, phospholipolysis	sPLA2	Group IIA sPLA2	122 aa, 13.8 kDa	monomer		[40]
BaltCRP	*B. alternatus* (Urutu pit viper)	K^+^-blocker, Immune stimulation	CRiSP		24.4 kDa	monomer		[104]
Baptides 1, 2 and 3	*B. arietans* (puff adder)	Postsynaptic neurotoxin		peptides	7 or 10 aa	monomer	Acetylated N-terminus, without Cys residues	[100]
Bitanarin	*B. arietans* (puff adder)	Phospholipolysis, Postsynaptic neurotoxin	sPLA2	Group II sPLA2	27.4 kDa	monomer	14 disulfides within a single chain	[59]
BomoTx	*B. moojeni* (Brazilian lancehead pit viper)	Myotoxin, induce pain, unable to phospholipolysis	sPLA2	sPLA2 of IIA group	122 aa, 13.84 kDa		Lys substitutes Asp49 in active center	[74]
α-Bungarotoxin	*B. multicinctus* (many-banded krait), Elapidae	α-neurotoxin	TFT	long-chain α-neurotoxin	76 aa, 8.2 kDa	monomer	Additional fifth disulfide in loop II	
Bothropstoxin-I	*B. jararacussu* (Jararacussu)	Myotoxin, Ca^2+^-channel blocker?	sPLA2	sPLA2 of IIA group	121 aa, 13.72 kDa	monomer	Lys substitutes Asp49 in active center	[49]
β-Bungarotoxins	*Bungarus spp*., (kraits) Elapidae	β-neurotoxin	sPLA2	Bungarotoxin	120aa + 60 aa, ~20 kDa	basic IA group sPLA2 subunit disulfide bound to KSPI-like subunit		
Calciseptine	*Dendroaspis polylepis* (black mamba) Elapidae	L-type Ca^2+^ channel blocker	TFT	Short-chain TFT	60 aa, 7.0 kDa			
Catrin-1 and catrin-2	*C. atrox* (Western diamondback rattlesnake)	K^+^-blocker	CRiSP		121 aa, 24.7 kDa	monomer	Differ by ionic strength at elution from a cation-exchange column	[107]
α-Colubritoxin	*C. radiatus* (radiated ratsnake), Colubridae	α-neurotoxin	TFT		77 aa, 8.5 kDa		N-terminally blocked by a pyroglutamic acid residue	[86]
Crotamine	*C. durissus terrificus* (South American rattlesnake)	Analgetics; K^+^-blocker	β-defensin	small basic myotoxins	42 aa, 4.9 kDa	monomer		[112,113,114,115,116,117,118,119,120,121]
Crotoxin	*C. durissus terrificus* (South American rattlesnake)	β-neurotoxin, phospholipolysis, anti-nociception	sPLA2	sPLA2 of IIA group	CB of 122 residues; CA of 88 residues	basic CB subunit of (CBa, CBb, Cbc, CBd) non-covalently bound to acidic CA subunit (CA1-CA4)	CA subunit consists of 3 covalently linked chains (α, 39 residues, β, 35 residues, and γ, 14 residues)	[35,40,42,54,55,56,61,63,64,65,66,67,68,69,70]
Dendrotoxins	*Dendroaspis spp*.(mambas), Elapidae	block voltage-dependent K^+^-channels	KSPI		57–60 aa			
DNTx I; DNTx-III	*D. russelli* (Russell’s viper)	α-neurotoxins	TFT	Short-chain TFT	6675 Da; 6849 Da	monomer	Cytotoxin-related; α-neurotoxin-related	[77,78]
Emunarecins EM1 and EM2	*E. multisquamatus* (saw-scaled viper)	Anticoagulants, Presynaptic neurotoxicity	C-type lectin-like proteins		70–160 kDa	Multimers of heterodimers	Composed of α and β subunits of 13 kDa and 18 kDa	[101]
Helicopsin	*Helicops angulatus* (Brown-banded watersnake), Colubridae		CRiSP		~20 kDa	monomer		[105]
HDP-2	*V*. *nikolskii* (Nikolsky’s viper)	Phospholipolysis, β-neurotoxin (?)	sPLA2	sPLA2 of IIA group	122 aa + 122 aa 13.7 + 13.8 kDa	acidic subunit non-covalently bound to basic subunit	Non-enzymatic acid subunit is inhibitor of basic subunit	[71]
Lys49 sPLA2s	*Agkistrodon, Bothrops, Trimeresurus* spp.	Myotoxin, induce pain, β-neurotoxin, unable to phospholipolysis	sPLA2	sPLA2 of IIA group	About 13–14 kDa or 27–28 kDa	Monomers or dimers	Lys substitutes Asp49 in active center	[48,73]
Piscivorin	*A. piscivorus piscivorus* (Northern cottonmouth)	K^+^-blocker	CRiSP		221 aa, 24.8 kDa	monomer		[107]
Rusvikunin	*D. russelii russelii* (Russell’s viper)	Anticoagulant, possible neurotoxicity	KSPI		6.9 kDa + 7.1 kDa	heterodimer	Composed of two KSPIs, rusvikunin and rusvikunin-II	[110]
Sarafotoxins, S6a-d	*Atractaspis* spp., Atractaspididae, Colubroidea	Activators of endothelin type A and B receptors	endothelin	peptides	15–30 aa; 21 aa, ~2500 Da	monomer	Have two disulfides. 4 major isopeptides S6a-d are composed of 21 aa	[123,124,125,126,127]
TFT-AF; TFT-VN,	*A. feae;* *V. nikolskii*	α-neurotoxins	TFT	non-conventional α-neurotoxin	68–70 aa	monomer	Additional fifth disulfide in loop I	[83]
Triflin	Habu snake *(Trimeresurus flavoviridis, P. flavoviridis*)	K^+^-blocker	CRiSP		221 aa, 24.8 kDa	monomer		[106]
Trimucrotoxin	*P. mucrosquamatus* (brown-spotted pit viper)	Presynaptic neurotoxin	sPLA2	Group IIA sPLA2	122 aa	monomer	Asn6-containing sPLA2	[53]
U1-viperitoxin-Dr1a	*D. russelii* (Russell’s viper)	Phospholipolysis, presynaptic neurotoxin	sPLA2s	Group IIA sPLA2	13.6 kDa	monomer		[24]
VaaChi	*V. ammodytes ammodytes* (nose-horned viper)	Putative K^+^-blocker	KSPI		7.5–7.6 kDa	monomer	Mix of 6 closely related isoforms	[111]
Vipoxin	*V. ammodytes ammodytes* (nose-horned viper)	β-neurotoxin, presynaptic neurotoxin?	sPLA2	Group IIA sPLA2				[51]
VRV-PL-IIIc, VRV-PL-VII, VRV-PL-IX	*D. russelii* (Russell’s viper)	Phospholipolysis, Pre- and postsynaptic neurotoxins	sPLA2	Group IIA sPLA2	13.0 kDa; 13.1 kDa; 12.5 kDa	monomer		[62]
Vurtoxin; Vur-PL	*V. ursinii* (meadow viper)	Phospholipolysis, anti-coagulant, Postsynaptic neurotoxin	sPLA2s	Group IIA sPLA2	122 aa, 13.9 kDa;	monomer		[61]
Vur-S49	*V. ursinii* (meadow viper)	Postsynaptic neurotoxin, unable to phospholipolysis	sPLA2	Group IIA sPLA2	122 aa, 13.9 kDa	monomer	Ser substitutes Asp49 in active center	[61]
Waglerins	*T. wagleri*	α-neurotoxin, GABA_A_ receptor modulation	Waglerins	Peptide from pre-pro region of the C-type natriuretic peptide	22–24 aa, 2.7–2.8 kDa	monomer	Basic proline-rich peptides with 1 disulfide	[90,91,92,93,94,95,96]

## Data Availability

Not applicable.
